# LncRNA HCG18 upregulates TRAF4/TRAF5 to facilitate proliferation, migration and EMT of epithelial ovarian cancer by targeting miR-29a/b

**DOI:** 10.1186/s10020-021-00415-y

**Published:** 2022-01-04

**Authors:** Fan Zhang, Bai-Hua Luo, Qi-Hui Wu, Qing-Ling Li, Ke-Da Yang

**Affiliations:** 1 Department of Gynecology, Xiangya Hospital, Central South University, Changsha, 410008 Hunan Province People’s Republic of China; 2 Department of Physiology, School of Basic Medical Science, Central South University, Changsha, 410008 Hunan Province People’s Republic of China; 3 Department of Pathology, Xiangya Hospital, Central South University, No. 87 Xiangya Road, Kaifu District, Changsha, 410008 Hunan Province People’s Republic of China

**Keywords:** lncRNA HCG18, Epithelial ovarian cancer, TRAF4, TRAF5

## Abstract

**Background:**

Although long noncoding RNA HLA complex group 18 (lncRNA HCG18) has been suggested to regulate cell growth in several tumours, the function of HCG18 in epithelial ovarian cancer (EOC) and its mechanism are still unclear.

**Methods:**

shRNAs were applied to reduce HCG18 and related genes. For overexpression of miRNA, a miRNA mimic was transfected into cells. Quantitative real-time PCR (qRT–PCR) was used to detect levels of HCG18, miR-29a/b, and mRNAs. MTT, colony formation, wound healing and Transwell assays were used to evaluate cell proliferation, migration and invasion, respectively. A luciferase reporter assay was utilized to evaluate NF-κB activity and the binding of miRNAs with HCG18 or TRAF4/5. BALB nude mice injected with cells stably expressing shHCG18 or shNC were used for in vivo modelling. Subcutaneous tumour growth was monitored in nude mice, and immunohistochemistry (IHC) was used to determine expression of the proliferation marker Ki67.

**Results:**

Abnormal expression of HCG18 and miR-29a/b was observed in EOC tissues. Knockdown of HCG18 using shRNA inhibited proliferation, migration, EMT and the proinflammatory pathway in EOC cells. miR-29a/b mimics and TRAF4/5 knockdown exhibited effects similar to HCG18 knockdown. Further experiments suggested that HCG18 directly targets miR-29a/b and upregulates TRAF4/5 expression, which are inhibited by targeting miR-29a/b. Moreover, overexpression of TRAF4/5 antagonized the inhibitory effect of HCG18 knockdown, suggesting that they are involved in HCG18-mediated oncogenic effects. Silencing HCG18 reduced tumour size and levels of Ki67 and TRAF4/5 while increasing miR-29a/b levels in vivo.

**Conclusions:**

Taken together, our data revealed an oncogenic signalling pathway mediated by HCG18 in ovarian cell lines, which functions as a ceRNA of miR-29a/b and thus derepresses expression levels of TRAF4/5, facilitating NF-κB pathway-mediated promotion of EOC cell proliferation and migration.

**Supplementary Information:**

The online version contains supplementary material available at 10.1186/s10020-021-00415-y.

## Background

Tumours can be viewed as wounds that never heal and are infiltrated by various inflammatory and immune cells (Coffelt and Visser 2014; Coussens and Werb 2002; Mantovani et al. 2008). The epithelial-to-mesenchymal transition (EMT) can lead to the loosening of cell–cell adherence complexes, which in turn facilitates the migration and invasion of cancer cells during metastatic cascades (Zhang and Weinberg 2018; Diepenbruck and Christofori 2016). As a consequence, cancer cells that undergo EMT are more aggressive and resistant to apoptosis (Kalluri and Weinberg 2009). Proinflammatory factors are also generated by the EMT in cancer cells, and inflammation can induce EMT in tumours (Long et al. 2016). Therefore, these two processes play essential roles in the metastasis of cancer cells.

As the most common type of ovarian cancer, epithelial ovarian cancer (EOC) accounts for more than 80% of all cases (George et al. 2016). However, the underlying mechanism of EOC proliferation and EMT remains largely elusive. Therefore, a better understanding of the mechanism and more investigation are required to improve the efficiency of EOC treatment. Most patients diagnosed with EOC exhibit extensive and distant metastasis, which plays a pivotal role in the prognosis of EOC and constitutes 80–90% of all EOC-related mortality (Rath et al. 2010; Saini et al. 2017; Wang et al. 2012). Recent studies have suggested that many molecules, including noncoding RNAs, may induce EMT in EOC cells through activation of several repressors of the epithelial phenotype, especially E-cadherin (Davidson et al. 2012; Gou et al. 2014; Koutsaki et al. 2014; Wang et al. 2018a; Wang et al. 2018b). Cytokines are also reported to be actively involved in EOC, and cytokine profiling suggests a complex network of cytokine expression exists in EOC (Saini et al. 2017; Apte et al. 2006; Clevenger et al. 2012; Matte et al. 2012; Vitolo et al. 1992).

Various studies have suggested that HCG18 is involved in a variety of human diseases, including diabetic peripheral neuropathy, intervertebral disc degeneration and cancer (Ren et al. 2021; Xi et al. 2017; Zhu 2021). A previous study reported that HCG18 was highly overexpressed in lung adenocarcinoma and could facilitate tumour growth (Li et al. 2020a). HCG18 expression is markedly enhanced in gastric cancer tissues, and knockdown of HCG18 suppresses the growth and metastasis of tumour cells (Liu et al. 2020). These studies demonstrated that HCG18 represents a potential biomarker for tumour therapy. Therefore, it is necessary to explore the mechanism and function of HCG18 in EOC.

Increasing evidence has shown that lncRNAs function as competitively endogenous RNAs (ceRNAs) to modulate signalling pathways by sponging miRNAs in cancer development (Chen et al. 2019; Kong, et al. 2018; Yuan et al. 2019; Zhu, et al. 2018). A previous study showed that miR-29a/b inhibits EOC (Zheng et al. 2018). However, the downstream targets of miR-29a/b in EOC have rarely been studied, and the mechanism of miR-29a/b’s function is still unclear. Considering that HCG18 acts as a ceRNA to absorb multiple miRNAs and plays a role in tumour cells (Ren et al. 2021; Li et al. 2020b), we investigated whether there is a targeting relationship between HCG18 and miR-29a/b.

The family of tumour necrosis factor receptor-associated factors (TRAFs 1–7) regulates signalling pathways of diverse receptors (Vitolo et al. 1992; Abdullah et al. 2018; Chen et al. 2018; Gentileschi et al. 2017; Shi and Sun 2018), and their expression levels vary in cancers. Several signalling pathways that are dependent on TRAF4/5 have been suggested to be involved in cancer, with evidence from studies of cultured human cancer cells or their xenografts in immunodeficient mice and patient samples. For example, TRAF4 has been reported to be involved in the TRAF4-AKT/NF-κB-Glut1/HK2/RSK4/Slug pathway, which regulates the proliferation, migration and EMT of various cancer cells, such as lung and breast cancer (Kim et al. 2017; Liu et al. 2018; Zhu et al. 2018). However, the function of TRAF4/5 in EOC cells has rarely been reported.

In this study, expression levels of HCG18 and miR-29a/b in EOC tissues and EOC cells were evaluated. We further investigated the function and signalling pathway of HCG18 and miR-29a/b in the growth and EMT of EOC cells. We discovered that HCG18 functions as a ceRNA of miR-29a/b and that miR-29a/b directly targets downstream TRAF4/5, which further regulates the AKT/-NF-κB pathway in cytokine release and EMT in EOC cells.

## Methods

### EOC specimens

A total of 30 EOC tissue specimens and 30 normal epithelial ovarian tissue specimens were collected from patients who underwent surgical resection at the Department of Gynaecology, Xiangya Hospital, Central South University, from 2017 to 2019. Two pathologists independently confirmed the tumour specimens, which were stored at  − 80 °C until analysis. This study was approved by the Ethics Committee of Xiangya Hospital, Central South University, and all specimens were handled and anonymized according to ethical and legal standards. Written informed consent was obtained from all patients.

### Cell culture

The cell lines CAOV3, OVCAR3, and SKOV3 were obtained from American Type Culture Collection (ATCC, USA), the OVSAHO cell line was obtained from Japanese Collection of research bioresources cell bank, the SNU119 cell line was obtained from Korean cell bank, the A2780 cell line and the normal human ovarian epithelial cell line IOSE80 were purchased from Yubo Biotech (Shanghai), and the immortalized normal human fallopian tube epithelial cell line FTE 187 was obtained from Jinsong Liu’s Laboratory. ES-2 is a cell line of ovarian clear cell adenocarcinoma, OVSAHO, SUN119, OVCAR3 and CAOV3 cells are high-grade ovarian serous adenocarcinoma, A2780 is a cell line of ovarian endometrioid adenocarcinoma, and SKOV3 is a cell line of well-differentiated adenocarcinoma. All cells were cultured in Dulbecco’s modified Eagle’s medium (HyClone, Logan, UT, USA) supplemented with 10% foetal bovine serum (FBS) and penicillin/streptomycin (100 U/mL). Cells were incubated at 37 °C in a 5% CO_2_ atmosphere.

### Lentivirus construction and transfection of shHCG18, miR-29 mimics and shTRAF4/5

Short hairpin RNAs targeting HCG18 (shHCG18; 5′-UUGGCUUCAGUCCUGUUCAUCAG-3′) and a negative control (shNC; 5′-AAUUCUCCGAACGUGUCACGU-3′) were purchased from GenePharma Company (Shanghai, China) and subcloned into the pLKO.1 vector. The pLKO.1-shHCG18 plasmid was then transfected into HEK293T cells with the psPAX2 packaging plasmid and pMD2.G envelop plasmid lentivirus. Cells (2 × 10^6^ cells per well) were cultured in 6-well plates to 80–90% confluence and then transfected with lentivirus (titre: 5 × 10^7^ TU/mL) at a multiplicity of infection (MOI) of 50 mixed with 5 μg/mL polybrene (Sigma–Aldrich, St. Louis, MO, United States) for 48 h. Mimics of miR-29a-3p and miR-29b-3p and negative control, TRAF4/5 overexpression and TRAF4/5 knockdown shRNAs and their negative control were purchased from GenePharma Company (Shanghai, China) and transiently transfected into the indicated cell lines. All cell transfection assays were performed using Lipofectamine 3000 (Invitrogen, Eugene, OR, USA).

### MTT assay

EOC cells (3 × 10^3^/well) were seeded into 96-well plates and assayed at 0 h, 24 h, 48 h, and 72 h after incubation with 25 μL of 4 mg/mL MTT solution (Solarbio, Beijing, China). For transfection, cells were first transfected with shHCG18, miR-29 mimics, shTRAF4/5, or vectors in 100 mm dishes and then split into 96-well plates. After incubating with MTT solution at 37 °C for 4 h, the solution was removed, dissolved in 150 μL dimethyl sulfoxide and subjected to 15 min of shaking. Absorbance was determined using a microplate spectrophotometer (BioTek Instruments, Winooski, VT, USA) at 490 nm.

### Colony formation assay

After being treated with shHCG18, miR-29 mimics, shTRAF4/5, or vectors, cells were subjected to trypsinization, and 1500 viable cells were subcultured in six-well plates. After culture for 2 weeks, the media were removed, and cells were fixed in 95% ethanol for 20 min. Then, the cells were stained with 0.5% crystal violet for 20 min. The colonies were subsequently quantified.

### Scratch wound healing assays

EOC cells were incubated in 6-well plates to ~ 80% confluence, and a scratch in the cell layer was generated using a pipette tip. PBS was then used to wash the plates to remove the scraped cells. Images were obtained 24 h later.

### Cell invasion assay

EOC cells harvested in serum-free medium were seeded in upper Transwell chambers (pore size, 6 μm; Corning Inc., Corning, NY, USA) after treatment with shHCG18, miR-29 mimics, shTRAF4/5, or vectors. Regular medium supplemented with 10% FBS was added to the bottom chamber. After incubation at 37 °C for 24 h, cells on the upper membrane were removed using a cotton swab, and the cells on the bottom surface of the membrane were fixed in 4% paraformaldehyde. Then, the cells were stained at room temperature with crystal violet for 15 min. Light microscopy was employed to quantify the number of cells to assess cell invasion.

### Total RNA extraction and real-time PCR

Total RNA was isolated using TRIzol reagent (Invitrogen), and the concentration was measured using a NanoDrop Spectrophotometer (Thermo Fisher Scientific, Waltham, MA, USA). A TaqMan® miRNA reverse transcription kit (Applied Biosystems, Foster City, CA, USA) was used for miRNA qPCR, with cDNA synthesized from 5 ng of total RNA. For the other genes, random primers from the RT Master Mix kit (Takara, Dalian, China) were used to synthesize cDNAs from total RNA. SYBR Green Real-Time PCR Master Mix (Toyobo, Osaka, Japan) and the ABI 7500 Sequence Detection system (Life Technologies, Grand Island, NY, USA) were used to perform qRT–PCR. Relative expression levels were normalized to that of GAPDH mRNA or U6. All experiments were performed in triplicate.

### Western blotting

Total proteins were extracted from EOC cell lines using RIPA buffer, and a BCA protein assay kit was used to determine the corresponding concentrations. Protein (25 μg) was isolated using 8% SDS–PAGE and subsequently transferred to PVDF membranes. BSA (1%) in TBS buffer was used to block the membranes, and primary antibodies were incubated at 4 °C. The membrane was then washed with 1 × TBST and incubated with a secondary antibody conjugate in 1 × TBS at room temperature for 1 h. The membrane was washed with 1 × TBST, and protein expression was determined using SuperSignal West Pico Chemiluminescent Substrate (Pierce Biotechnology). The loading control was β-actin detected on the same blot. The primary antibodies were purchased from Cell Signaling Technology as follows: β-actin (8H10D10), Vimentin (D21H3), E-cadherin (24E10), MMP-2 (D4M2N), MMP-9 (D6O3H), ZEB1 (D80D3), NF-κB p65 (D14E12), Slug (C19G7), TWIST1 (#46702), Snail (C15D3), Phospho-NF-κB p65 (Ser536) (93H1), acetyl-NF-κB p65 (Lys310) (D2S3J), AKT (pan) (C67E7), phospho-AKT Substrate (RXXS*/T*) (110B7E), β-tubulin (9F3), lamin A/C (4C11), TRAF4 (D1N3A), and TRAF5 (D3E2R).

### Bioinformatics and dual-luciferase reporter assay

The binding sites between HCG18 and the 3′ UTRs of TRAF4/5 mRNA with miR-29a/b were predicted using starBase (http://starbase.sysu.edu.cn). A QuikChange Mutagenesis kit (Stratagene) was then used to generate the mutations. The 3′-UTR sequences of HCG18, TRAF4 or TRAF5 containing wild type or mutated binding sites were subcloned into the pRL-TK luciferase reporter. All constructs were sequenced to verify integrity. EOC cells were transfected with 300 ng of firefly luciferase reporter and 25 ng of Renilla luciferase plasmid plus 900 ng of empty vector or miRNA mimic plasmid. Twenty-four hours after transfection, luciferase assays were performed using a Dual Luciferase Reporter Assay kit (Promega), and the ratio of firefly to Renilla luciferase activity was determined.

For NF-κB transcriptional activity, cells were cotransfected with 100 ng of the pNFκB reporter luciferase plasmid. Renilla plasmid (Promega) containing 5 ng pRL-TK was also transfected as a control signal. All transfections were performed using Lipofectamine 3000 (Invitrogen). The signals were detected using a Dual Luciferase Reporter Assay Kit (Promega).

### Tumour xenografts

Female BALB/c-nude mice (20–22 g, 6 weeks old) were purchased from the Animal Experiment Centre of the Chinese Academy of Sciences (Shanghai, China) and used as xenograft animal models (n = 16). Animals were raised in specific pathogen-free conditions, and all animal experiments were approved by the animal ethics committee of Xiangya Hospital, Central South University. Mice were randomly divided into 2 groups, and CAOV3 cells, (5 × 10^6^) stably expressing shHCG18 or shNC, were injected subcutaneously into the right flank of nude mice under aseptic conditions. Then, we measured tumour size using callipers every 4 days, and after 24 days, mice were sacrificed. Then, xenograft tumour tissues were harvested for immunohistochemical staining. Then, the remaining tissues were stored in a − 80 °C freezer for analysis of mRNA and protein levels.

### Immunohistochemistry (IHC)

Tumour tissue sections were blocked in 4% non-fat milk containing 1% Triton X-100, incubated with Ki67 primary antibody (1:200, ab16667, Abcam) at 4 °C overnight and then washed with PBS 3 times. Next, after incubation with the biotinylated secondary antibody for 30 min at room temperature, the sections were stained with freshly prepared 3,3′-diaminobenzidine (DAB), followed by counterstaining.

### Statistical analysis

Data are shown as the mean and standard deviation (SD). Student’s t-test was used to compare differences between two groups for continuous variables. One-way analysis of variance (ANOVA) followed by Tukey’s post hoc test was used for multiple comparisons. All analyses were performed using GraphPad Prism 6 (GraphPad Software, Inc.) P < 0.05 was considered statistically significant.

## Results

### Expression levels of HCG18, miR-29a and miR-29b in tumour tissues

**Fig. 1:**
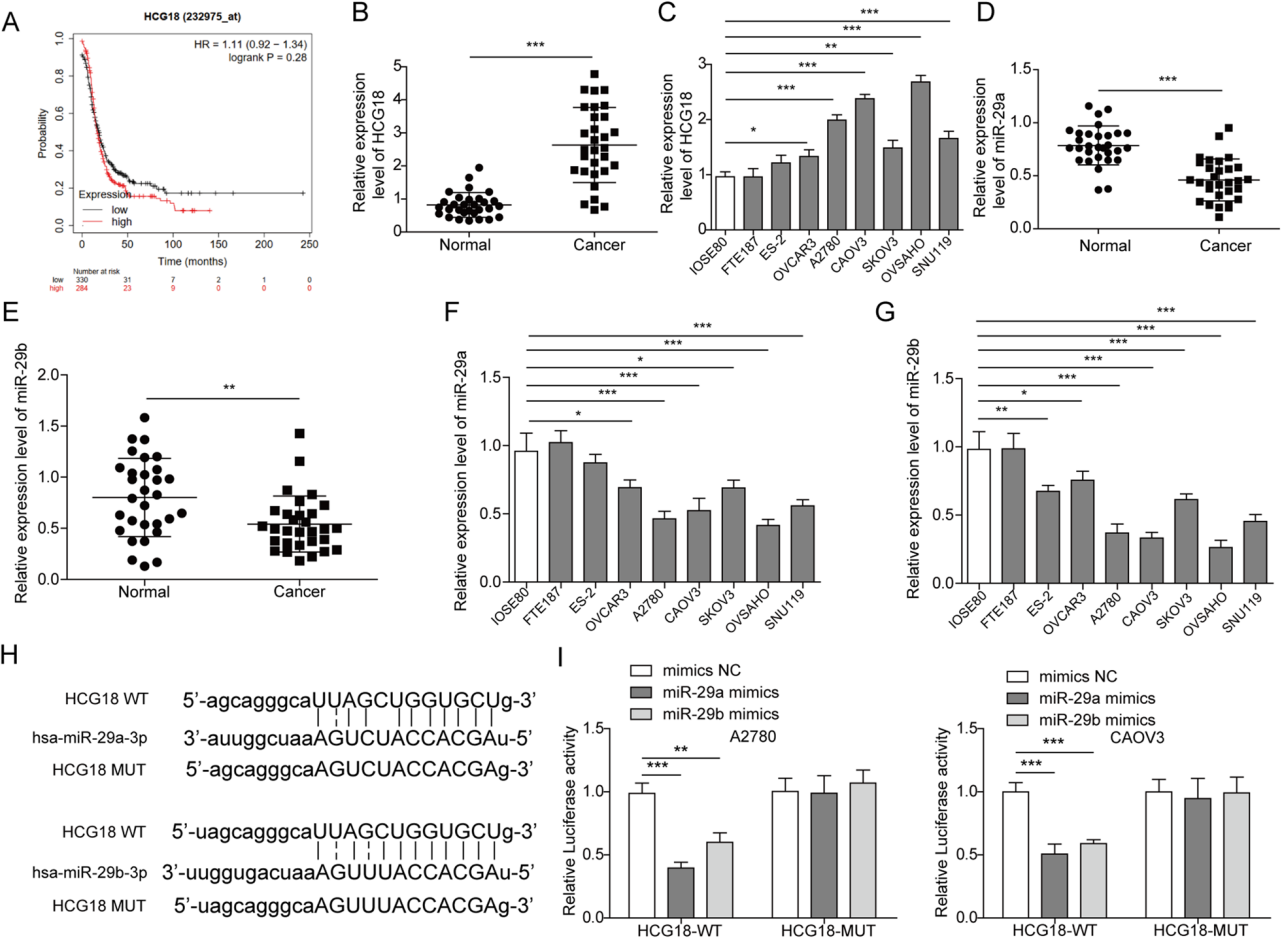
Expression levels of HCG18 and miR-29a/b in patient tumour tissues and EOC cell lines. **A** The data about the correlation analysis of HCG18 levels and the survival of patients with EOC was downloaded from Kaplan Meier plotter. **B** HCG18 expression levels in EOC tissues (n = 30) and normal tissues (n = 30) as detected by qRT–PCR. **C** HCG18 expression levels in EOC cell lines and normal cell lines by qRT–PCR (n = 3). **D**–**E** Expression levels of miR-29a/b in EOC tissues (n = 30) and normal tissues (n = 30) was detected using qRT–PCR. **F**–**G** Expression levels of miR-29a/b in EOC cell lines and normal cell lines by qRT–PCR (n = 3). **H**–**I** The interaction of HCG18 and miR-29a/b was determined using sequence analysis and dual luciferase reporter assays (n = 3). *P < 0.05, **P < 0.01, ***P < 0.001

Data from Kaplan Meier plotter (http://kmplot.com/analysis/index.php?p=background) indicated that high expression of HCG18 might be relevant with poor prognosis in EOC (Fig. 1A). Then, expression levels of HCG18, miR-29a and miR-29b in EOC tissues were assessed by qRT–PCR. Normal ovarian tissue specimens were used as healthy controls. As shown in Fig. 1B, HCG18 levels in tumour tissues (n = 30) were increased compared to those in normal tissues (n = 30). The correlations between HCG18 expression levels and clinicopathological features in human EOC specimens were analysed (Table 1). We found that HCG18 expression level in EOC tissues was significantly higher in high tumour stage (III–IV) and tumour grade (G3). Moreover, HCG18 was significantly high-regulated in EOC patients with lymph node metastasis compared to those without metastasis. While, HCG18 expression was not correlated with other clinicopathological features, including age, histological subtype. We then assessed HCG18 levels in several types of EOC cells and observed similarly higher expression of HCG18 compared to the normal ovarian epithelial cell line IOSE80 and FTE187 (Fig. 1C). In contrast, miR-29a and miR-29b were downregulated in both the tumour tissues (Fig. 1D, E) and EOC cell lines (Fig. 1F, G).

**Table 1:** Correlation between HCG18 expression and clinicopathological features of epithelial ovarian cancer tissues

Clinical parameters	HCG18 expression		P value
	High (n = 15)	Low (n = 15)	
Age (years)			
< 50	8	6	0.715
≥ 50	7	9	
Histological subtype			
Serous	12	7	0.128
Other	3	8	
FIGO stage			
I–II	4	12	0.009
III–IV	11	3	
Histological grade			
G1 + G2	2	10	0.008
G3	13	5	
Lymph node metastasis			
Absent	6	12	0.060
Present	9	3	

Bioinformatics analysis revealed that HCG18 binds with miR-29a/b (Fig. 1H). When cotransfecting miR-29a/b mimic or miR-NC EOC cells together with the luciferase construct containing either the WT or MUT binding sequence of HCG18, we observed that the miR-29a/b mimic specifically and significantly decreased the luciferase activity containing the WT but not MUT HCG18 binding sequence (Fig. 1I), indicating a direct interaction between HCG18 and miR-29a/b.

### Silencing HCG18 inhibits the proliferation, migration, and invasion of EOC cells

**Fig. 2:**
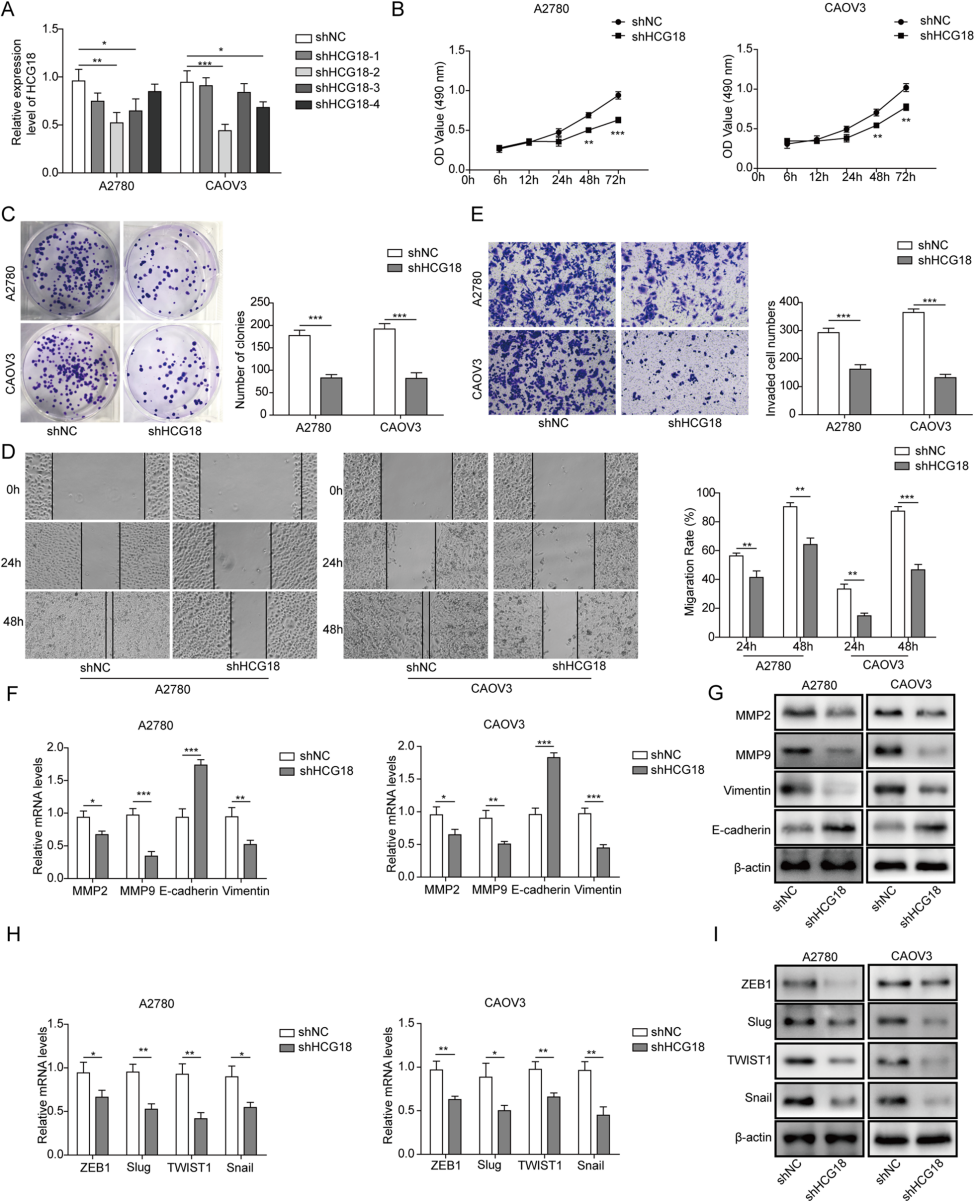
Effect of HCG18 knockdown on EOC cell proliferation, migration, invasion and EMT. **A** Screening of shRNA for the knockdown of HCG18 in EOC cells. **B**, **C** Effect of HCG18 knockdown on EOC cell proliferation as determined by MTT and colony formation assays. **D** Effect of HCG18 knockdown on EOC cell migration as determined by scratch wound healing assay. **E** Effect of HCG18 knockdown on EOC cell invasion as detected by Transwell assay. **F**, **G** Effect of HCG18 knockdown on expression of EMT markers as measured by qRT–PCR and Western blot. **H**, **I** Effect of HCG18 knockdown on the expression of EMT transcription factors as determined by qRT–PCR and Western blot. n = 3. *P < 0.05, **P < 0.01, ***P < 0.001

To explore the effect of HCG18 expression on cell proliferation, migration and invasion, we tested four shRNAs to knock down HCG18 in EOC cells (A2780 and CAOV3). shRNA#2, which effectively reduced the expression of HCG18 by 40–50% in both cell lines (Fig. 2A), was used for the knockdown of HCG18 in subsequent studies.

First, cell viability was evaluated using MTT. We observed that compared to control shRNA, HCG18 knockdown inhibited cell viability in both cell lines (Fig. 2B). The colony formation assay in Fig. 2C clearly showed that HCG18 knockdown reduced the number of colonies after seeding by almost 50%. Second, the influence of HCG18 on cell migration was examined using a wound healing assay, and the results revealed that cell migration was repressed by shHCG18 but not shNC (Fig. 2D). Finally, in Fig. 2E, cell invasion ability was evaluated using the Transwell assay. Similarly, HCG18 knockdown with shHCG18 significantly attenuated the invaded cell number compared to that of the negative control. Overall, these results indicated that HCG18 knockdown inhibits the proliferation, migration, and invasion of EOC cells.

EMT is primarily identified in the context of normal cell differentiation during early development (Chaffer et al. 2016; Jolly et al. 2017; Pastushenko and Blanpain 2018). Here, we chose E-cadherin, MMP2, MMP9, and Vimentin as biomarkers for EMT in EOC. As shown in Fig. 2F, mRNA levels of E-cadherin were increased by shHCG18, while those of MMP2, MMP9, and Vimentin were reduced. The Western blot results in Fig. 2G also showed a similar effect on the protein expression levels of these genes. We further tested the effect of HCG18 knockdown on mRNA and protein levels of EMT-related transcription factors, including the ZEB1, Slug, TWIST1 and Snail (Fig. 2H, I). The results revealed that HCG18 knockdown reduced expression levels of all of these factors involved in the EMT process. Taken together, these results suggested that HCG18 is crucial for EMT in EOC cells and that knockdown of HCG18 effectively inhibits EMT.

### HCG18 facilitates cytokine release via the AKT-NF-κB pathway and TRAF4/5

**Fig. 3:**
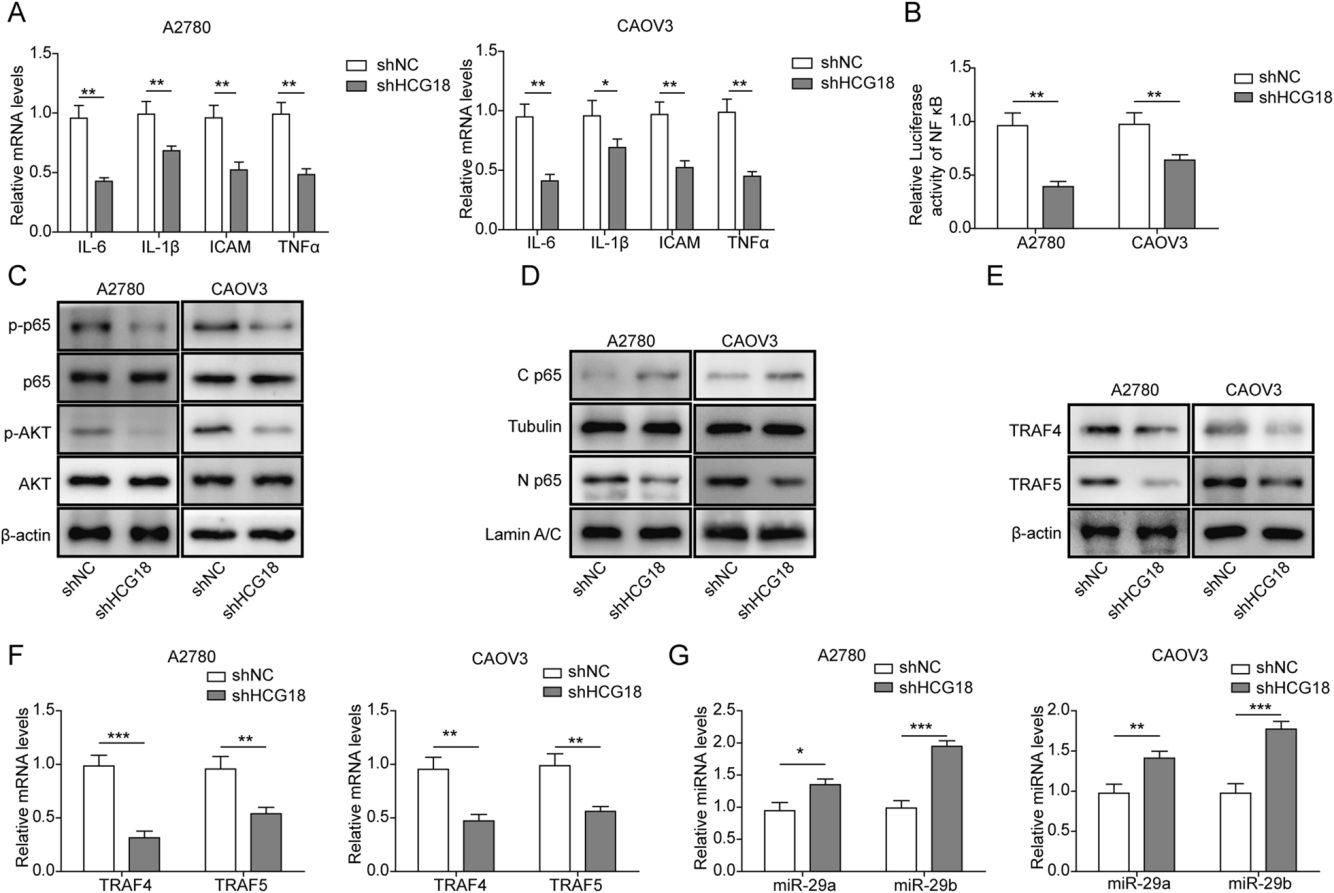
Effect of HCG18 knockdown on the proinflammatory process and HCG18 targeting of miR-29a/b. **A** Effect of HCG18 knockdown on cytokines as determined by qRT–PCR. **B** HCG18 knockdown reduced NF-κB activity, as shown by luciferase assays. **C**, **D** Effect of HCG18 knockdown on NF-κB and AKT signalling pathways by Western blot. **E**, **F** HCG18 knockdown reduced TRAF4/5 expression in EOC cells, as determined by Western blot and qRT–PCR. **G** HCG18 knockdown increased miR-29a/b levels in EOC cells, as determined by qRT–PCR. n = 3. *P < 0.05, **P < 0.01, ***P < 0.001

Inflammation has been shown to play an oncogenic role in the tumour growth of EOC (Matte et al. 2012; Kacinski 1995; Li and Ni 2000; Tavares Murta et al. 1999; Terranova and Rice 1997). EOC cells continuously secrete cytokines such as IL-6, IL-1β, and TNF-α (Kolomeyevskaya et al. 2015). They stimulate oncogenicity in both autocrine and paracrine fashions and receive signals from the tumour microenvironment (TME) (Kuninaka et al. 2000). We assessed expression levels of these cytokines through qRT–PCR, and the results showed that HCG18 knockdown reduced expression levels of these cytokines (Fig. 3A), suggesting the importance of HCG18 in the inflammation process in EOC.

The NF-κB pathway has been characterized as a signalling cascade involved in prototypical proinflammation, largely based on its role in regulating the expression of proinflammatory genes, including cytokines, chemokines, and adhesion molecules (Abdullah et al. 2018; Shi and Sun 2018; Wu et al. 2018). We observed reduced transcriptional activity in response to HCG18 knockdown (Fig. 3B). Prior studies have reported that AKT modulates the transcriptional activity of NF-κB by simulating phosphorylation and degradation of IκB (Wang et al. 2012). Importantly, phosphorylation of p65 and AKT was reduced in both cell lines treated with shHCG18 (Fig. 3C), indicating the involvement of HCG18 in the AKT/NF-κB pathway and its proinflammatory effect. Activation of the NF-κB transcription factor has been linked with nuclear translocation of the p65 component of the complex. We also found that knockdown of HCG18 suppressed the nuclear translocation of p65 by Western blot (Fig. 3D).

TRAF4/5 are important intermediates of GITR-induced NF-κB activation (Esparza and Arch 2004). Our results showed that HCG18 knockdown reduced the expression levels of TRAF4/5 in EOC cells at both the mRNA and protein levels (Fig. 3E, F). Therefore, we investigated whether HCG18 affects miR-29a and miR-29b by qRT–PCR. As shown in Fig. 3G, inhibition of HCG18 increased both miR-29a and miR-29b levels in EOC cell lines (A2780 and CAOV3). However, the relationship between HCG18 and TRAF4/5 is unclear. In the following experiment, we examined whether HCG18 functions as a ceRNA of miRNA that derepresses the expression of TRAF4/5.

### Effect of miR-29a and miR-29b on the proliferation, migration, and invasion of EOC cells

To determine the effect of miR-29a/b on ovarian cells, we overexpressed miR-29a/b in A2780 and CAOV3 cells (Additional file 1: Fig. S1A). We then tested the miR-29a/b mimics for cell growth inhibition using MTT and colony formation assays (Additional file 1: Fig. S1B, C). The results showed that introduction of miR-29 by mimics exerted a cell growth inhibition effect similar to the knockdown of HCG18. The wound healing and Transwell assays for cell migration and invasion also yielded similar results (Additional file 1: Fig. S1D, E) in both cell lines. We further studied the effect of miR-29 mimics on EMT in EOC cell lines by measuring biomarkers of EMT and transcription factors as described above. Interestingly, the mimics again induced a similar effect as HCG18 knockdown (Additional file 1: Fig. S1F–I), suggesting that the same pathway may be involved for miR-29 and HCG18.

### miR-29a and miR-29b inhibit inflammation in ovarian cell lines

The increased expression of miR-29a and miR-29b in response to the mimics also exerted the same effect as HCG18 knockdown, affecting the AKT-NF-κB pathway (Additional file 2: Fig. S2B, S2C-D) and cytokine release (Additional file 2: Fig. S2A). These results indicate that miR-29a and miR-29b regulate the AKT-NF-κB pathway during proinflammatory activity in EOC cell lines.

### TRAF4 and TRAF5 are upregulated in EOC, and knockdown of TRAF4 and TRAF5 inhibits the proliferation, migration, and invasion of EOC cells

**Fig. 4:**
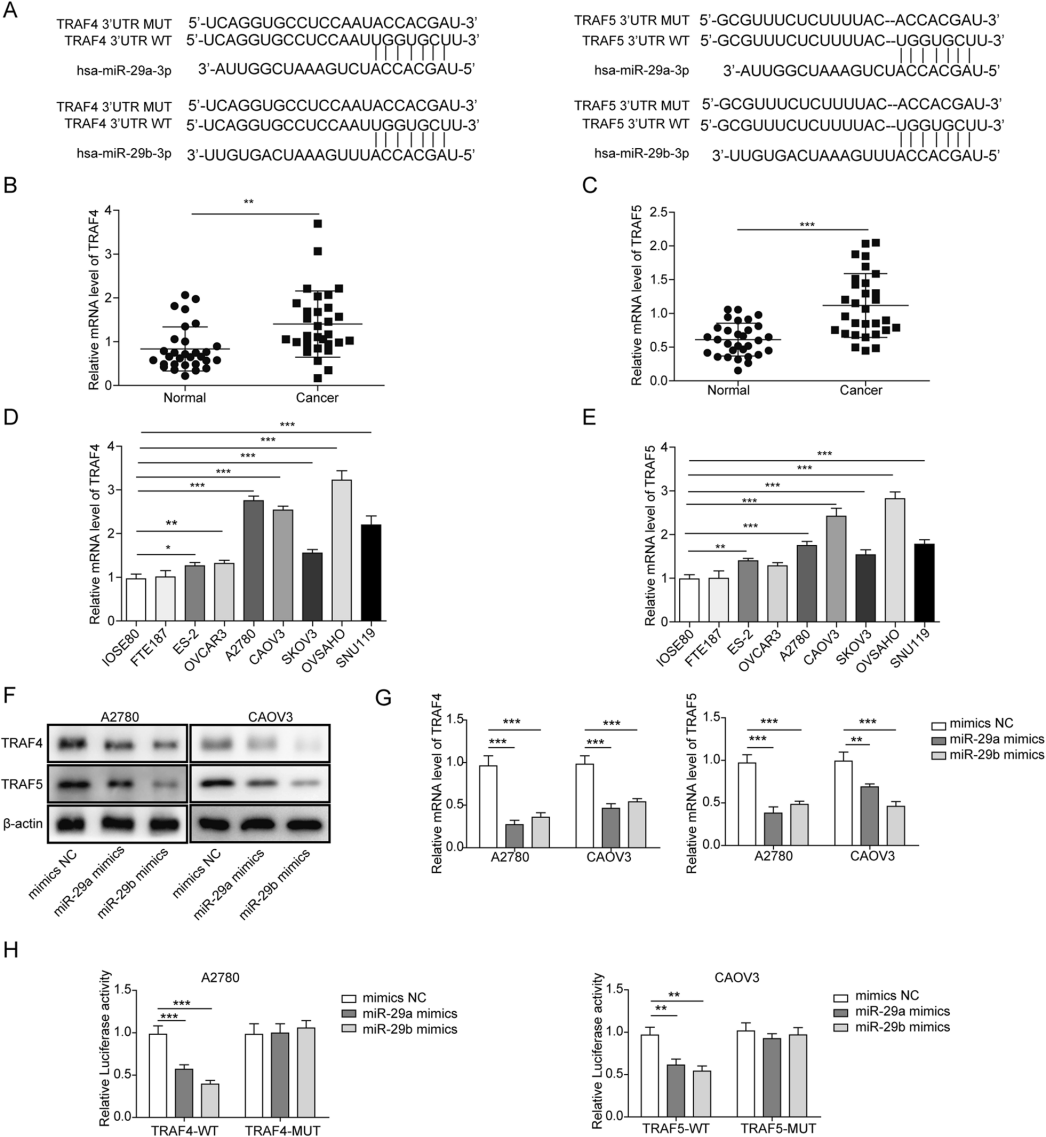
Expression of TRAF4/5 in vivo and in vitro. **A** The interaction of TRAF4/5 mRNA and miR-29a/b as determined by sequence analysis. **B**, **C** TRAF4/5 expression levels in EOC tissues (n = 30) and normal tissues (n = 30) as measured by qRT–PCR. (D-E) Expression levels of HCG18 in EOC cell lines and normal cell lines by qRT–PCR (n = 3). **F**, **G** miR-29a/b overexpression reduced TRAF4/5 expression in EOC cells, as determined by Western blot and qRT–PCR (n = 3). **H** The interaction of TRAF4/5 mRNA and miR-29a/b as determined by dual luciferase reporter assay (n = 3). *P < 0.05, **P < 0.01, ***P < 0.001

Previous reports have suggested that the miR-29 family targets TRAF4 and TRAF5. We analysed the sequences of miR-29a-3p and miR-29b-3p and found that they have direct targeting sequences in the 3′-UTRs of TRAF4 and TRAF5 (Fig. 4A). Next, we analysed TRAF4 and TRAF5 expression in the 30 EOC tissues and 20 normal tissues described above. The qRT–PCR experiments indicated that TRAF4 and TRAF5 mRNA levels were relatively higher in the tumour tissues (Fig. 4B, C). We also tested several EOC cell lines and observed increased expression of TRAF4 and TRAF5 in these cell lines (Fig. 4D, E). We found that miR-29a/b mimics reduced TRAF4/5 protein (Fig. 4F) and mRNA (Fig. 4G) levels in A2780 and CAOV3 cell lines, suggesting the inhibitory effect of miR-29a/b on TRAF4/5 expression. Luciferase reporter assays validated that both miR-29a and miR-29b mimics reduced the luciferase activity of wild type TRAF4/5, while reporters containing mutated TRAF4/5 binding sequences were not influenced by miR-29a/b, suggesting direct binding of miR-29a/b to TRAF4/5 (Fig. 4H). These results strongly suggested that expression of TRAF4 and TRAF5 is related to HCG18 and miR-29 in EOC cell lines.

Therefore, we tested whether knockdown of TRAF4 and TRAF5 expression by shRNA exerted similar effects to HCG18 knockdown and miR-29 mimics. First, we confirmed the downregulation of TRAF4 and TRAF5 by shTRAF4 and shTRAF5 in EOC cell lines A2780 and CAOV3, respectively (Additional file 3: Fig. S3A). MTT and colony formation assays demonstrated that both shTRAF4 and shTRAF5 inhibited the growth of ovarian cells (Additional file 3: Fig. S3B, C). Wound healing and Transwell assays revealed the inhibitory effect of shTRAF4 and shTRAF5 on cell migration and invasion, which was similar to shHCG18 and miR-29 mimics (Additional file 3: Fig. S3D, E). We further tested the effect of shTRAF4 and shTRAF5 on expression of EMT biomarkers and transcription factors using qRT–PCR and Western blot (Additional file 3: Fig. S3F–I). Interestingly, TRAF4/5 knockdown again showed similar effects to HCG18 knockdown and miR-29 mimics, suggesting that the same pathway may be involved for TRAF4/5, miR-29a/b and HCG18.

Next, we examined TRAF4 and TRAF5 in the inflammatory pathway. TRAF4 and TRAF5 inhibited the expression and release of inflammatory cytokines (Additional file 4: Fig. S4A, B). However, TRAF4 and TRAF5 exerted different functions in the AKT-NF-κB pathway. TRAF4 knockdown inhibited the phosphorylation of AKT, while TRAF5 did not, suggesting that TRAF4 promotes AKT phosphorylation, while TRAF5 directly regulates the activation of NF-κB signalling (Additional file 4: Fig. S4C–E). Overall, these data strongly suggested that TRAF4/5 are direct targets of miR-29 in the process of proinflammatory activity in EOC cell lines.

### Overexpression of TRAF4 and TRAF5 antagonizes the effect of HCG18 knockdown

**Fig. 5:**
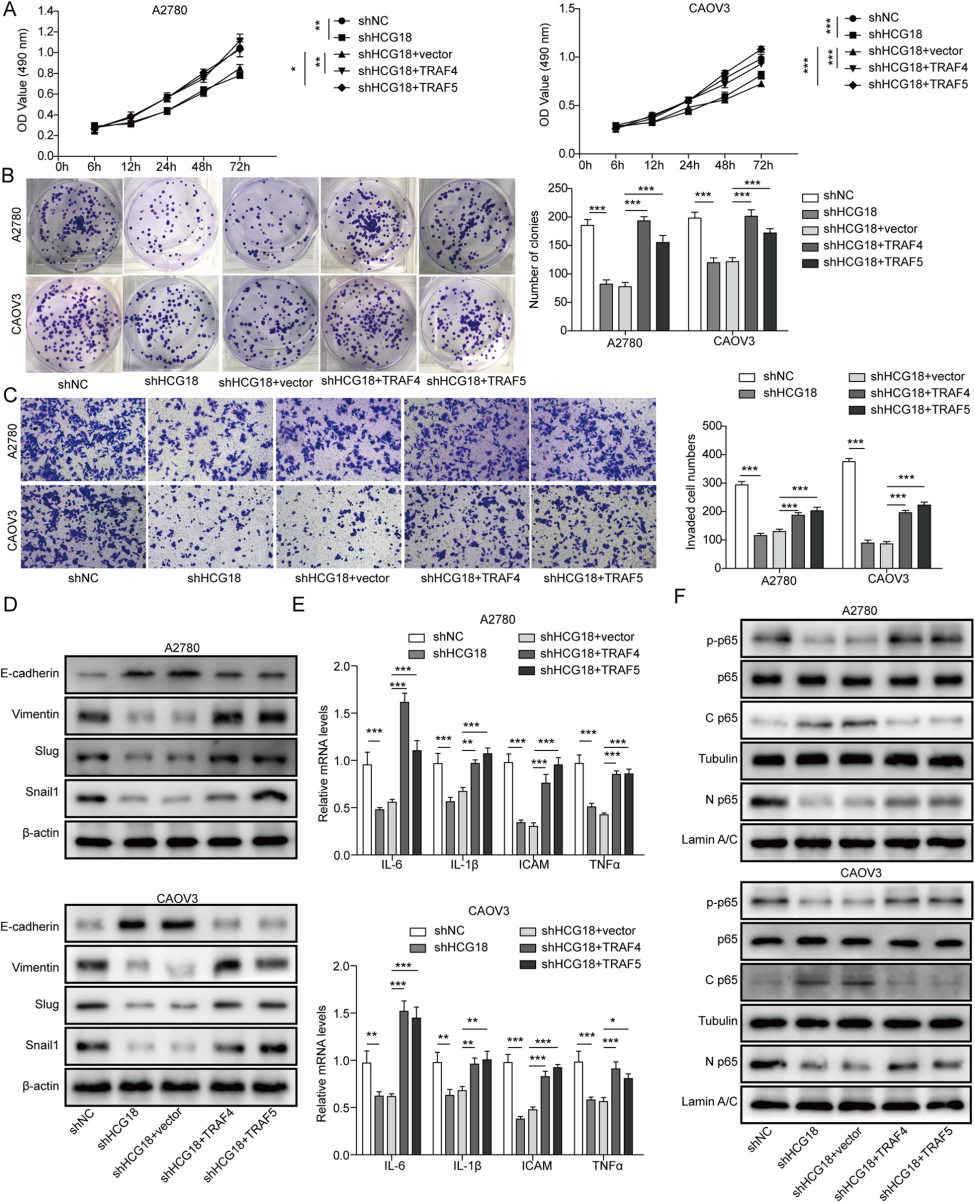
TRAF4/5 overexpression antagonizes the effect of HCG18 knockdown in EOC cells. **A**, **B** TRAF4/5 overexpression recovered the EOC cell proliferation inhibition effect of HCG18 knockdown, as determined by MTT and colony formation assays. **C** Effect of TRAF4/5 overexpression and HCG18 knockdown on EOC cell invasion as determined by Transwell assay. **D** Effect of TRAF4/5 overexpression and HCG18 knockdown on expression levels of EMT markers and transcription factors as detected by Western blot. **E** Effect of TRAF4/5 overexpression and HCG18 knockdown on cytokines as determined by qRT–PCR. **F** Effect of TRAF4/5 overexpression and HCG18 knockdown on the NF-κB and AKT signalling pathways by Western blot. n = 3. *P < 0.05, **P < 0.01, ***P < 0.001

To further validate the association of TRAF4/5 with HCG18, we treated A2780 and CAOV3 EOC cells with shHCG18 and TRAF4/5 expression vectors simultaneously. We found that the suppressive effect of HCG18 knockdown on the proliferation and migration of EOC cells was reversed in response to TRAF4 and TRAF5 overexpression (Fig. 5A–C). We further tested downstream pathways, including EMT biomarkers, EMT transcription factors, NF-κB, and cytokines. As shown in Fig. 5D, cotransfection of TRAF4/5 expression vectors generally restored the effect of HCG18 knockdown on EMT biomarkers and EMT transcription factors in both A2780 and CAOV3 cell lines. Similarly, overexpression of TRAF4 and TRAF5 reversed HCG18 shRNA-inhibited mRNA levels of proinflammatory cytokines (Fig. 5E) and activation of NF-κB (Fig. 5F). All of these results suggested that overexpression of TRAF4/5 by vectors significantly antagonizes the effect of shHCG18, implying that HCG18 promotes EOC progression by regulating TRAF4/5 expression.

### Further analysis in OVSAHO cell lines

**Fig. 6:**
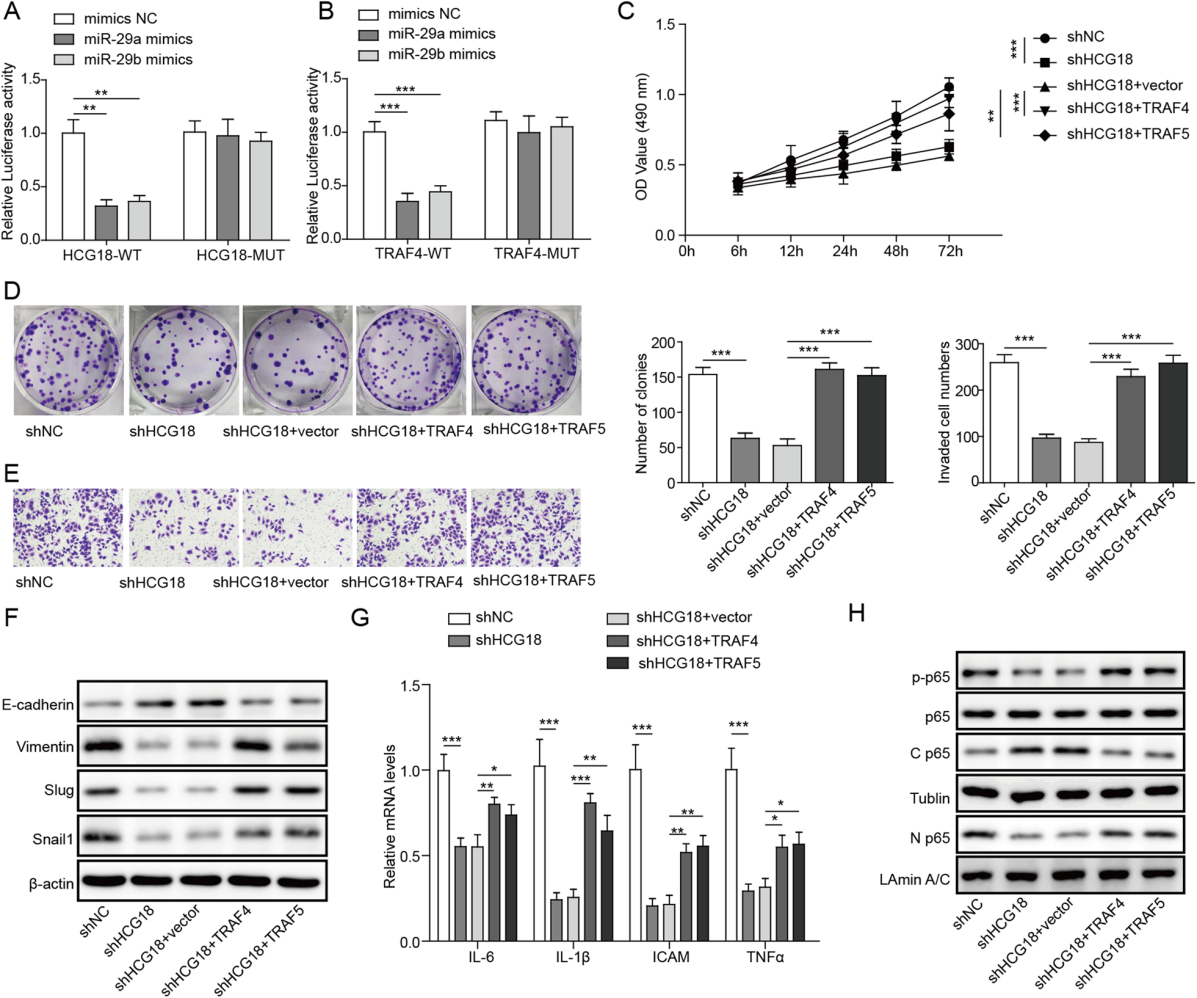
Further analysis in OVSAHO cell lines. **A**, **B** The interaction of HCG18 and miR-29a/b, as well as of TRAF4/5 mRNA and miR-29a/b was determined by dual luciferase reporter assay in OVSAHO cells. OVSAHO cells were transfected with shHCG18, shHCG18 + TRAF4/5 plasmids, then the cell proliferative ability (**C**, **D**), cell migration capacity (**E**), the expression of EMT markers and transcription factors (**F**, **G**), and the levels of cytokines (**H**) were measured by MTT, colony formation, Transwell, Western blot and qRT-PCR, respectively. n = 3. *P < 0.05, **P < 0.01, ***P < 0.001

To obtain more reliable results *in vitro*, we verified parts of important above conclusions in OVSAHO cells. Firstly, the dual-luciferase reporter assay indicated that there was direct interaction between HCG18 and miR-29a/b, as well as between miR-29a/29b and TRAF4/5 in OVSAHO cell line (Fig. 6A, B). Subsequently, plasmids encoding shHCG18 and TRAF4/5 were transfected into OVSAHO cells. The results showed that overexpression of TRAF4/5 blocked the inhibitory effect of HCG18 knockdown on the proliferation and migration of OVSAHO cells (Fig. 6C–E). As expected, the effect of HCG silencing on EMT biomarkers and EMT transcription factors was restored by overexpressing TRAF4/5 in OVSAHO cells (Fig. 6F, G). The downregulation of proinflammatory cytokines induced by HCG18 knockdown was reversed by TRAF4/5 overexpression (Fig. 6H). Taken together, we further confirmed the regulatory effect of HCG18 on the expression of miR-29a/29b and TRAF4/5, and the cell biological functions in OVSAHO cell line.

### Knockdown of HCG18 inhibits the tumorigenicity of EOC cells in nude mice

**Fig. 7:**
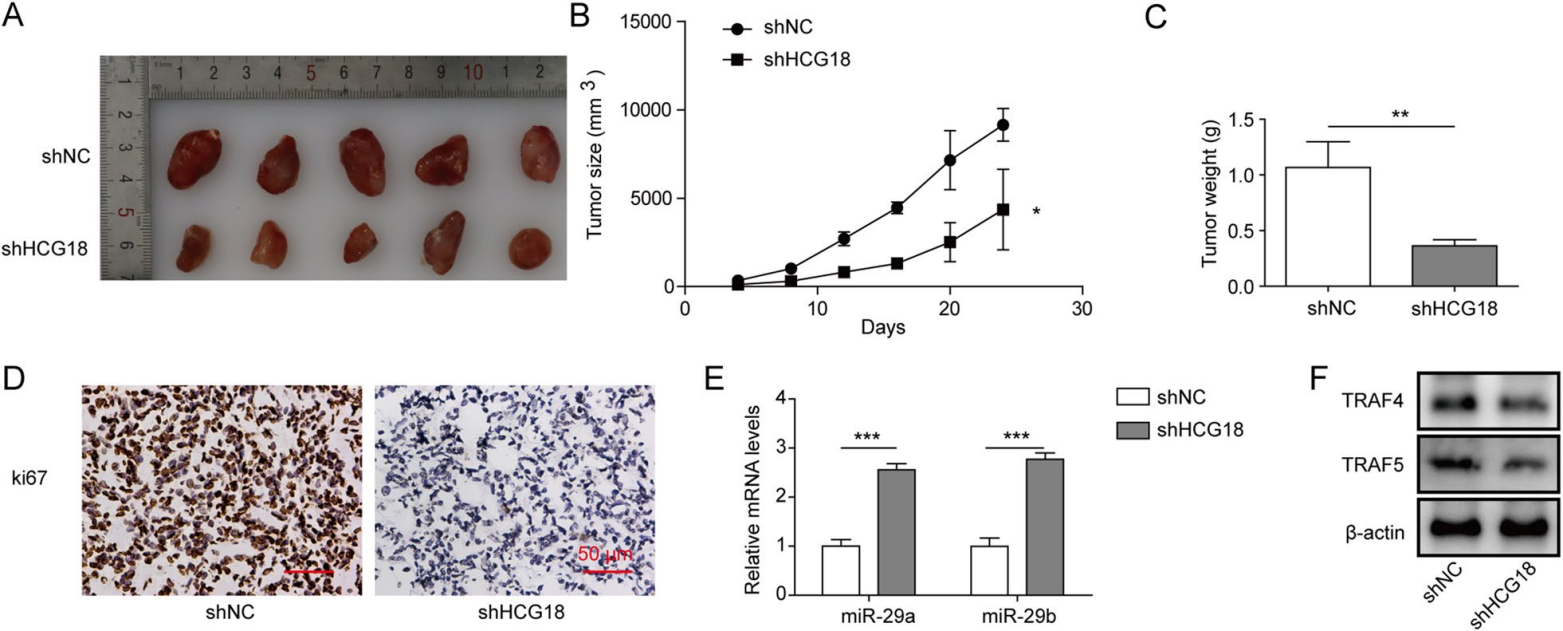
Effect of HCG18 knockdown on the tumorigenicity of EOC cells in nude mice. CAOV3 cells infected with shHCG18 or shNC were subcutaneously injected into the right flank of nude mice. **A**–**C** HCG18 knockdown inhibited tumour size and weight. **D** Expression of Ki67 was decreased after silencing HCG18. Scale bar = 50 μm. **E**, **F** miR-29a/b was upregulated while TRAF4/5 was downregulated after silencing HCG18. n = 5. *P < 0.05, **P < 0.01, ***P < 0.001

To investigate whether the effects of HCG18 are effective in vivo, CAOV3 cells stably expressing shHCG18 expression vectors were subcutaneously injected into nude mice. We observed that tumour size in the shHCG18 group displayed significant growth inhibition (Fig. 7A–C). Furthermore, the IHC results showed that knockdown of HCG18 induced low expression of Ki67 (Fig. 7D). qRT–PCR and Western blot results indicated that expression of miR-29a/b was upregulated, while TRAF4/5 levels were decreased after silencing HCG18 (Fig. 7E, F). Taken together, these results indicate that the HCG18/miR-29a/b/TRAF4/5 pathway might also play a role in vivo, which requires more experimental data to confirm.

## Discussion

Our experimental results suggested that HCG18 promotes the inflammatory response, facilitates EMT of EOC, and ultimately increases proliferation, migration and invasion in vitro and in vivo. Mechanistically, HCG18 inhibits miR-29 a/b levels and upregulates the expression of TRAF4/5, which are direct targets of miR-29a/b.

HCG18 has been identified as a carcinogenic gene in several cancer types (Li et al. 2020a; Yang et al. 2021). For example, Li et al. demonstrated that knockdown of HCG18 repressed cell proliferation and metastasis in nasopharyngeal carcinoma progression (Li et al. 2019). Furthermore, a recent study demonstrated that HCG18 acts as a ceRNA for miR-152–3p, thereby derepressing its target gene DNAJB12 to promote the development of gastric cancer (Yang et al. 2021; Ma et al. 2020). Using starBase, we also identified a binding relationship between HCG18 and miR-152–3p. Moreover, expression levels of miR-152 were found to be dysregulated in ovarian cancer in previous studies (Li et al. 2018; Zhou et al. 2012; Qin et al. 2018), indicating a potential role of HCG18 in ovarian cancer. However, the expression and mechanism of HCG18 in EOC have not been reported, and the detailed function and regulatory pathway in particular need more exploration. Here, we illustrated the mechanism underlying the function of HCG18 in EOC. The effect of HCG18 overexpression on the inflammatory process in several diseases has also been demonstrated (Ren et al. 2021; Xi et al. 2017). However, it is unclear whether this is the same in cancer, particularly in EOC. To investigate the mechanism of HCG18 in EOC, we demonstrated here that HCG18 directly targets and inhibits miR-29a/b in EOC cells using luciferase reporter assays and evaluated its effect on downstream signalling pathways. It is well known that lncRNAs can target multiple miRNAs in cancer cells, and miR-29a/b is one of the target miRNAs of lncRNA HCG18. However, whether miR-29a/b is the top hit for HCG18 in ovarian cancer remains undetermined. We further revealed the regulation of cytokines and inflammatory pathways by HCG18 in cancer cells for the first time, which functions by inhibiting the expression of miR-29a/b as a ceRNA.

In particular, miR-29a was shown to repress epithelial EOC progression by directly targeting SIRT1. Here, we analysed the sequence of miR-29a/b and the 3′UTR of TRAF4/5, and the results suggested direct targeting of miR-29a/b on TRAF4/5 for the first time. The effect of miR-29a/b on TRAF4/5 affected activation of the AKT-NF-κB pathway and thus cytokine release and EMT. Therefore, for the first time, our study identified miR-29a/b as a new target in EOC cells and further demonstrated the involvement of the inflammatory pathway in the regulation of miR-29a/b on the proliferation and migration of EOC cells.

A completely different type of inflammation is that which occurs in tumour development (Coffelt and Visser 2014; Coussens and Werb 2002; Galdiero et al. 2018). Most solid malignancies exhibit an intrinsic inflammatory response to develop a protumorigenic microenvironment. Changes in the tumour microenvironment are induced by the recruitment of leukocytes and lymphocytes and the expression of tumour-promoting chemokines and cytokines (Rath et al. 2010; Andrieu et al. 2018; Molinaro et al. 2018; Moraes et al. 2018). In lung cancer cells, TRAF4 activates AKT through ubiquitination and is a candidate molecular target for the prevention and therapy of lung cancer (Kim et al. 2017; Zhu et al. 2018). Meanwhile, TRAF5 is directly involved in NF-κB activation and protection from cancer cell death, displaying a slightly different mechanism in the AKT-NF-κB pathway. Both TRAF4 and TRAF5 function as activators in the inflammatory process and promote cancer development. Our results further confirmed the function of TRAF4/5 in EOC cells. Identification of TRAF4/5 as targets of miR-29a/b in the signalling pathway of HCG18 explains the inhibition of HCG18 on EOC cell proliferation, migration and invasion.

A major challenge to EOC therapy is exploring the unique peritoneal tumour microenvironment in EOC progression and metastasis (Vlieghere et al. 2015). It was reported that in lung cancer cells, TRAF4 facilitates cancer development by modifying the tumour microenvironment in normal fibroblasts (Kim et al. 2017). Considering our data in EOC cells, HCG18 and TRAF4 are potential regulators of the tumour microenvironment in EOC. We speculated that HCG18 could affect the activity of cancer-associated fibroblasts and promote tumour development by affecting the tumour microenvironment. Overall, our study pioneered this field, and further investigations should be performed to fully reveal the function of HCG18 in EOC-associated fibroblasts and the tumour microenvironment.

## Conclusions

For the first time, our study thoroughly investigated the function of HCG18 in vitro and in vivo. We found that HCG18 promotes proliferation and migration of EOC through inflammatory pathways and EMT. We further discovered the important downstream targets miR-29a/b and TRAF4/5 and elucidated the effect of HCG18 on proliferation and migration. HCG18 promotes EOC development by directly inhibiting miR-29a/b and increasing the expression of TRAF4/5, which are the downstream targets of miR-29a/b. These findings provide insights into the function of lncRNAs in EOC development and uncover critical information for the diagnosis and therapy of EOC.

## Supplementary Information

**Additional file 1: Figure S1.** Effect of miR-29a/b overexpression on EOC cell proliferation, migration, invasion and EMT. (A) Screening of miR-29a/b mimics in EOC cells. (B-C) Effect of miR-29a/b mimics on EOC cell proliferation as determined by MTT and colony formation assays. (D) Effect of miR-29a/b mimics on EOC cell migration as determined by scratch wound healing assay. (E) Effect of miR-29a/b mimics on EOC cell invasion as determined by Transwell assay. (F) Effect of miR-29a/b mimics on expression of EMT markers as detected by qRT–PCR. (G) Effect of miR-29a/b mimics on expression of EMT markers as determined by Western blot. (H) Effect of miR-29a/b mimics on expression levels of EMT transcription factors as detected by qRT–PCR. (I) Effect of miR-29a/b mimics on the expression of EMT transcription factors as determined by Western blot. n=3. *P<0.05, **P<0.01, ***P<0.001.

**Additional file 2: Figure S2.** The signalling pathway of miR-29a/b overexpression in the proinflammatory process. (A) Effect of miR-29a/b overexpression on cytokines as determined by qRT–PCR. (B) miR-29a/b overexpression reduced NF-κB activity, as shown by luciferase assays. (C-D) Effect of miR-29a/b overexpression on NF-κB and AKT signalling pathways assessed by Western blot. n=3. **P<0.01, ***P<0.001.

**Additional file 3: Figure S3.** Effect of TRAF4/5 knockdown on EOC cell proliferation, migration, invasion and EMT. (A) shRNA significantly knocked down TRAF4/5 levels in EOC cells. (B-C) Effect of TRAF4/5 shRNA on EOC cell proliferation as determined by MTT and colony formation assays. (D) Effect of TRAF4/5 shRNA on EOC cell migration as determined by scratch wound healing assay. (E) Effect of TRAF4/5 shRNA on EOC cell invasion as determined by Transwell assay. (F) Effect of miR-29a/b mimics on expression of EMT markers as measured by qRT–PCR. (G) Effect of miR-29a/b mimics on expression of EMT markers as determined by Western blot. (H) Effect of miR-29a/b mimics on expression of EMT transcription factors as determined by qRT–PCR. (I) Effect of miR-29a/b mimics on the expression of EMT transcription factors as determined by Western blot. n=3. *P<0.05, **P<0.01, ***P<0.001.

**Additional file 4: Figure S4.** Effect of TRAF4/5 knockdown on the proinflammatory signalling pathway. (A-B) Effect of TRAF4/5 shRNA on cytokines as determined by qRT–PCR. (C-E) Effect of HCG18 knockdown on the NF-κB and AKT signalling pathways by Western blot. n=3. *P<0.05, **P<0.01, ***P<0.001.

## Data Availability

The datasets used or analyzed during the current study are available from the corresponding author on reasonable request.

## Abbreviations

EMT: Epithelial-to-mesenchymal transition
EOC: Epithelial ovarian cancer
ceRNA: Competitively endogenous RNA
FBS: Fetal bovine serum
IHC: Immunohistochemistry
SD: Standard deviation
ANOVA: Analysis of variance
TME: Tumor microenvironment
