# Supplementary figures and images for: LncRNA HCG18 upregulates TRAF4/TRAF5 to facilitate proliferation, migration and EMT of epithelial ovarian cancer by targeting miR-29a/b
Source: Mol Med. 2022 Jan 4;28:2. doi: 10.1186/s10020-021-00415-y (PMC8725507; doi:10.1186/s10020-021-00415-y)

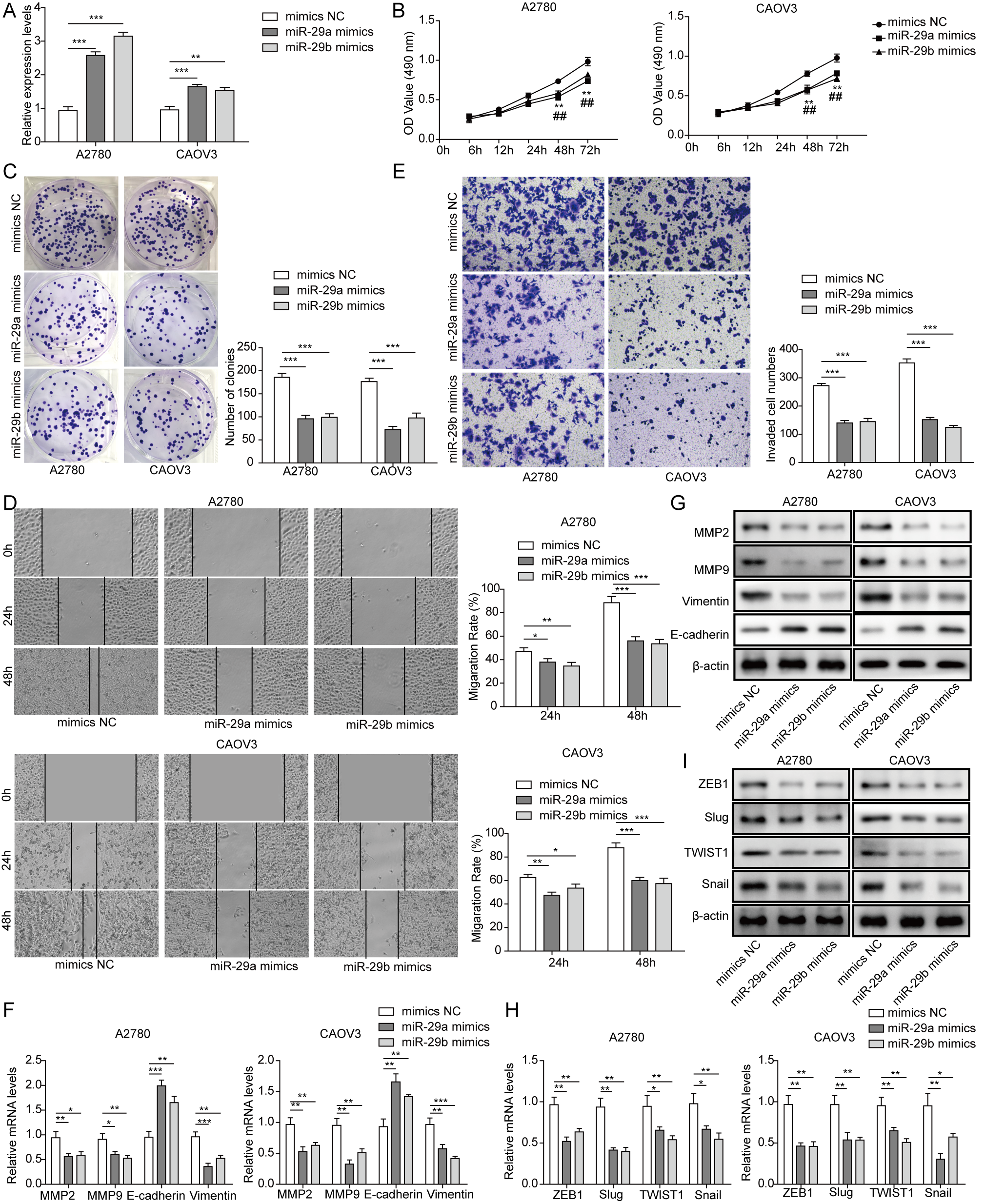

Supplement: Supplementary file 1 — Additional file 1: Figure S1. Effect of miR-29a/b overexpression on EOC cell proliferation, migration, invasion and EMT. (A) Screening of miR-29a/b mimics in EOC cells. (B-C) Effect of miR-29a/b mimics on EOC cell proliferation as determined by MTT and colony formation assays. (D) Effect of miR-29a/b mimics on EOC cell migration as determined by scratch wound healing assay. (E) Effect of miR-29a/b mimics on EOC cell invasion as determined by Transwell assay. (F) Effect of miR-29a/b mimics on expression of EMT markers as detected by qRT–PCR. (G) Effect of miR-29a/b mimics on expression of EMT markers as determined by Western blot. (H) Effect of miR-29a/b mimics on expression levels of EMT transcription factors as detected by qRT–PCR. (I) Effect of miR-29a/b mimics on the expression of EMT transcription factors as determined by Western blot. n=3. *P<0.05, **P<0.01, ***P<0.001. [file 10020_2021_415_MOESM1_ESM.tif]

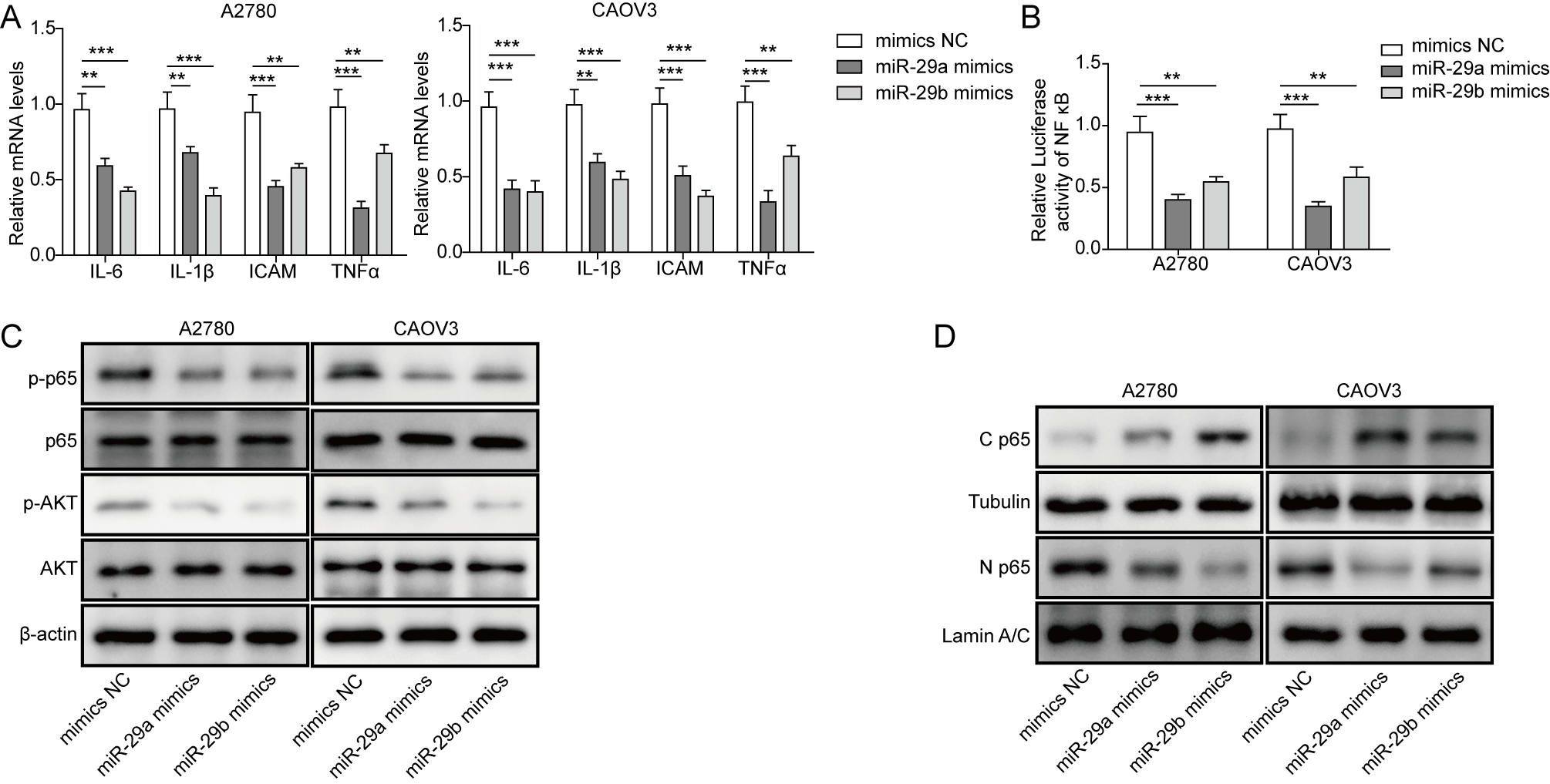

Supplement: Supplementary file 2 — Additional file 2: Figure S2. The signalling pathway of miR-29a/b overexpression in the proinflammatory process. (A) Effect of miR-29a/b overexpression on cytokines as determined by qRT–PCR. (B) miR-29a/b overexpression reduced NF-κB activity, as shown by luciferase assays. (C-D) Effect of miR-29a/b overexpression on NF-κB and AKT signalling pathways assessed by Western blot. n=3. **P<0.01, ***P<0.001. [file 10020_2021_415_MOESM2_ESM.tif]

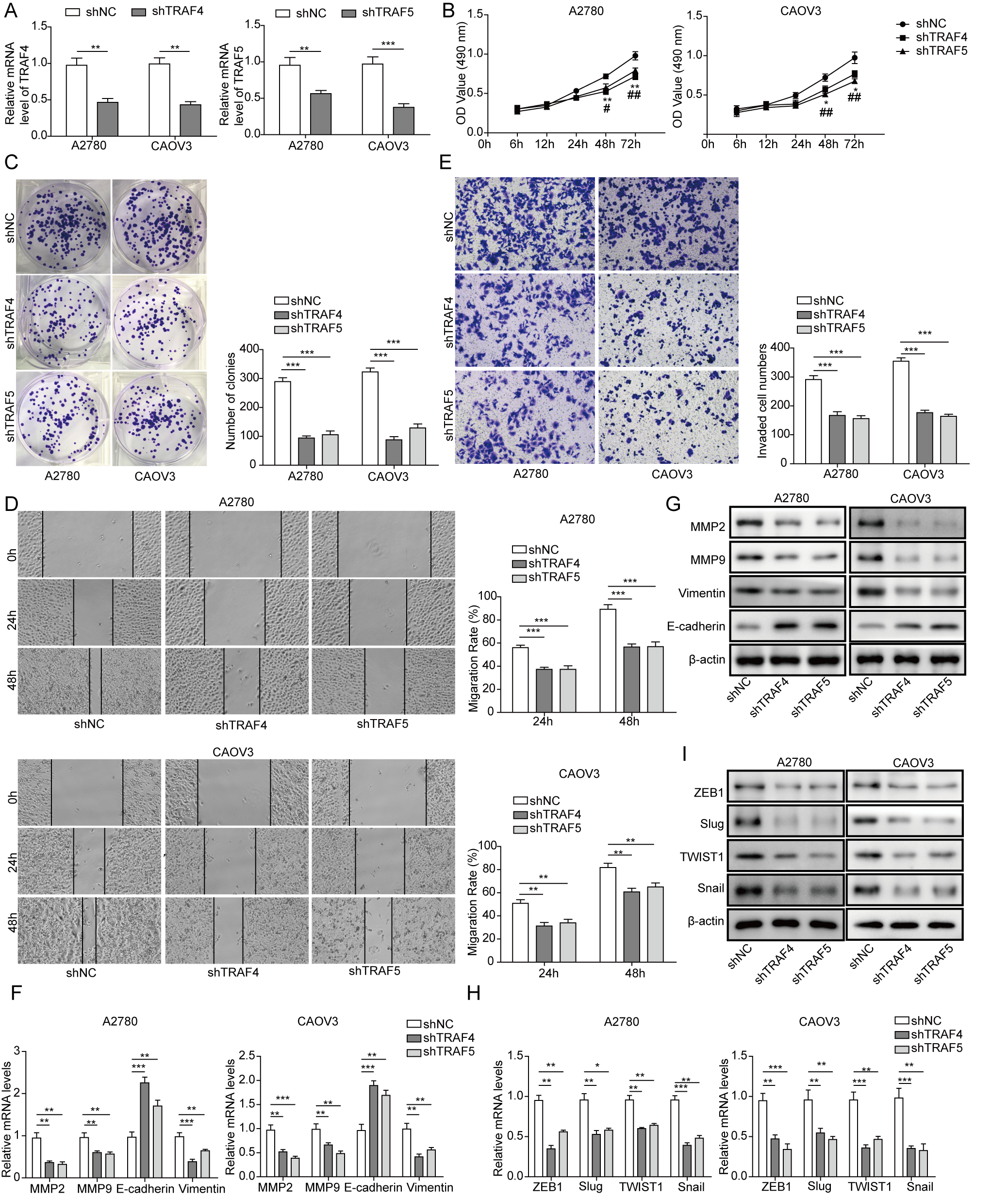

Supplement: Supplementary file 3 — Additional file 3: Figure S3. Effect of TRAF4/5 knockdown on EOC cell proliferation, migration, invasion and EMT. (A) shRNA significantly knocked down TRAF4/5 levels in EOC cells. (B-C) Effect of TRAF4/5 shRNA on EOC cell proliferation as determined by MTT and colony formation assays. (D) Effect of TRAF4/5 shRNA on EOC cell migration as determined by scratch wound healing assay. (E) Effect of TRAF4/5 shRNA on EOC cell invasion as determined by Transwell assay. (F) Effect of miR-29a/b mimics on expression of EMT markers as measured by qRT–PCR. (G) Effect of miR-29a/b mimics on expression of EMT markers as determined by Western blot. (H) Effect of miR-29a/b mimics on expression of EMT transcription factors as determined by qRT–PCR. (I) Effect of miR-29a/b mimics on the expression of EMT transcription factors as determined by Western blot. n=3. *P<0.05, **P<0.01, ***P<0.001. [file 10020_2021_415_MOESM3_ESM.tif]

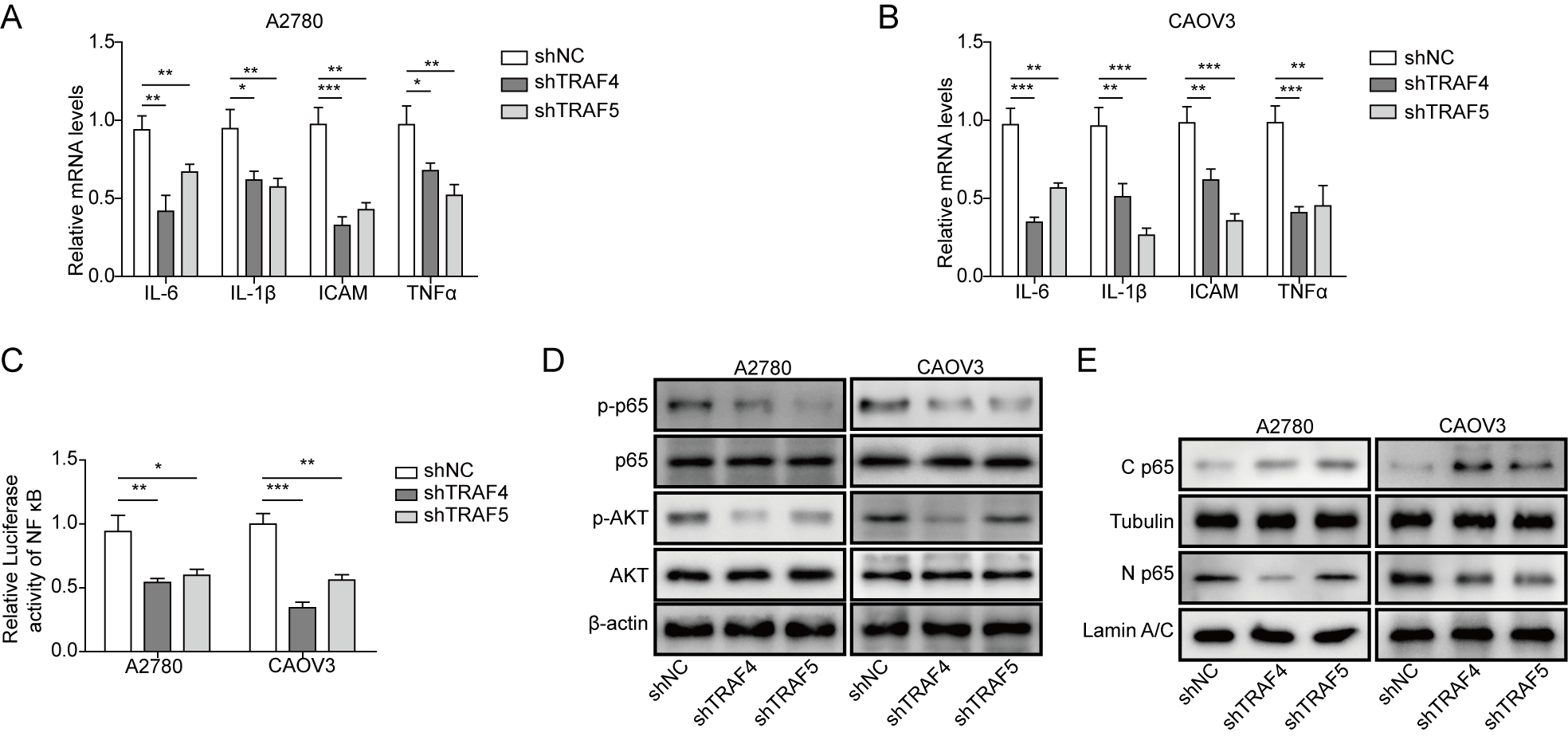

Supplement: Supplementary file 4 — Additional file 4: Figure S4. Effect of TRAF4/5 knockdown on the proinflammatory signalling pathway. (A-B) Effect of TRAF4/5 shRNA on cytokines as determined by qRT–PCR. (C-E) Effect of HCG18 knockdown on the NF-κB and AKT signalling pathways by Western blot. n=3. *P<0.05, **P<0.01, ***P<0.001. [file 10020_2021_415_MOESM4_ESM.tif]
